# Tracheotomy for the Removal of a Foreign Body

**Published:** 1877-03-20

**Authors:** R. Inge


### TRACHEOTOMY FOR THE REMOVAL OF A FOREIGN BODY.

BY R. INGE, M. D.

In September last, Horace Whitsell, a mulatto child, 2½ years of age, while eating a watermelon, was suddenly seized with a violent paroxysm of coughing attended with considerable dyspnœa. These violent symptoms passed off in a few minutes; but still there remained some difficulty in respiration, which at times became very alarming. On examining the patient a distinct thud could be heard about the throat when any effort at coughing was made, the cough being croupy. During the respiratory movements there was only a wheezing sound produced, as if the air was passing through a very small orifice. Being satisfied a foreign body was present, I advised an operation, but the parents being unwilling to have it done it was deferred until the 6th of October when the symptoms of asphyxia became so alarming that death seemed inevitable. I then requested my friend Dr. E. Young, to perform tracheotomy. The incision was made just below the isthmus of the thyroid gland. Three rings of the trachea being divided, a violent paroxysm of coughing ensued, but no foreign body was expelled. Owing to hemorrhage from the wound, all efforts at removing the body was stopped, and a tracheotomy tube inserted. Immediately the respiration became quiet and easy, and the little fellow commenced playing about the house as if nothing was the matter, presenting quite a contrast to his previous condition. This satisfied us that the foreign body was above the opening in the trachea, probably in the larynx.

In ten days the tube was removed, and an effort made at removing the seed. The coughing was most violent, but nothing was accomplished. We then closed the wound with a soft towel, but found that no air would pass through the larynx. A feather was then passed into the wound up into the larynx, when a very faint croupy cough resulted. Owing to hemorrhage around the wound the tube had to be replaced. In three days it was again removed when immediately a violent cough expelled the seed through the wound,

After which, closing the wound with a towel, a little air would pass through the larynx, but not enough to sustain life. The tube was replaced, for two days, when upon removal it was found that respiration could be performed by the natural channel. The wound in the neck healed in a week perfectly.

This is an interesting case from two points: first, the position of the seed, and the means we employed for dis–lodging. Secondly, the time that elapsed from the operation to the final expulsion of the seed.

